# Self-Exposure on Instagram and BMI: Relations With Body Image Among Both Genders

**DOI:** 10.5964/ejop.7221

**Published:** 2023-05-31

**Authors:** Desiré Abrante, Mónica Carballeira

**Affiliations:** 1 Department of Clinical Psychology, Psychobiology and Methodology, Faculty of Psychology and Speech Therapy, University of La Laguna, San Cristóbal de La Laguna, Santa Cruz de Tenerife, Spain; University of Wroclaw, Wroclaw, Poland

**Keywords:** self-exposure, BMI, body image, Instagram

## Abstract

Social media users can actively choose how they portray themselves and review the information they share to form and manage positive impressions on their audience. A high Body Mass Index (BMI) can lead to a bias of attention towards self-reported unattractive personal body areas. This dysfunctional body-related attention can lead to increased body dissatisfaction. Concerning social networks, people who usually post more photos on Instagram more frequently show higher body satisfaction. The main objective of this work was to analyze the relationship between BMI in young people, their own exposition on Instagram, positive body image and certain psychological variables: self-esteem, coping and well-being. The population-based sample consisted of 687 young Instagram users aged between 18 to 35 years old. The results found in this work revealed that BMI had a significant influence on the body exposure on Instagram in both genders, as well as in body image and certain psychological variables, such as coping and well-being. Moreover, we found that there is not a direct relation between BMI and the exposure of the entire body on Instagram. This relationship exists through positive body image, appearance care and management appearance behaviors. These results imply that positive body image affects body’s exposure, so people with obesity or overweight tend to upload less photos with half or full body visible than people with normal weight or underweight. This is not because of their weight, but their valuation and appreciation of their bodies.

Self-presentation is defined as the attempt by individuals to control impressions of themselves and therefore involves a conscious effort to enact behaviors by creating a desired persona for an audience. (Schlenker & Pontari, 2000). Social media users can actively choose how to portray themselves and review the information they share to form and manage positive impressions on their audience (Lee-Won et al., 2014). This observation of the image of oneself in photographs produces a visual attention directed to the body appearance that could trigger behaviors such as body monitoring, which may be related to body shame. Thus, within the context of social comparisons, a visual reminder of appearance and repeated opportunities for self-assessment is offered by the images of their bodies on social media (Butkowski et al., 2019). Furthermore, people who perceive a discrepancy between their appearance and an idealized body image may be ashamed of their body (Dakanalis et al., 2015).

Body mass index (BMI) is a measure of people’s weight in relation to their height. BMI has been shown to predict body image over time for both men and women, such that variations in BMI measurements over time continue to influence body perception and appearance (Holsen et al., 2012). In this line, BMI is related to most aspects of body image, increasing body dissatisfaction as BMI increases in both genders, although the relationship is stronger for women than for men (Ålgars et al., 2009). Thus, research shows that the affective and cognitive aspects of the construction of body image are considerably impaired in the obese population and this dissatisfaction with body image is related to low self-esteem (Piko et al., 2020), an increase in depression symptoms (Sarwer et al., 2005) and a lower quality of life (Wilson et al., 2013). In people with a high BMI, forms of maladaptive coping are also shown, such as self-distraction, self-blame, or avoidance, which are also linked to a negative body image, low self-esteem (Pinkasavage et al., 2015) and less social support (Yayan & Çelebioğlu, 2018).

Concerning body size, the research has suggested an attention bias paid towards self-reported unattractive personal body areas, due to a higher BMI. This dysfunctional body-related attention can cause an increment on body dissatisfaction (Couture Bue, 2020). However, actual body size may matter much less than a person's sense of satisfaction with their body size. People who are happy with a larger body size may be more likely to proudly share their own body than people who are slimmer but suffer from poor body image (Wagner et al., 2016). According to their research, people with a larger body size tend to report more body dissatisfaction, and both together were able to predict the number of selfies (Wagner et al., 2016). When it comes to gender, it has been found that female Instagram users tend to post a significant number of more selfies than men (Simerson, 2016). Additionally, women tend to upload more portrait photos than full-length photos to their social network’s profiles (Haferkamp et al., 2012).

The effects of BMI on the exposure of one's own body in social networks seem to be influenced by certain factors of individual differences; that is, not everyone is equally affected by their weight when it comes to uploading photos of themselves to the networks. Therefore, we consider a series of variables that have been shown to be related to exposure on social networks, such as body image, coping or well-being.

Body image is a multidimensional and dynamic construct that encompasses how we perceive, think, feel, and act towards our bodies, and forms a continuum that goes from healthy body perceptions (accurate and mostly positive) to unhealthy body perceptions (inaccurate and generally negative; Voelker et al., 2015). Cash et al. (2004) argued that the information received about physical appearance generated pressure and idealization of certain physical attributes. Concerning social networks, there is an increasing number of findings that indicate a positive link between the use of social networks and body image in young adults (Manago et al., 2015). For example, Instagram users who are most satisfied with their body image tend to make a greater number of publications of themselves, such as selfies (Butkowski et al., 2019).

Self-esteem is a concept that encompasses positive or negative evaluations of oneself, defined as the beliefs of the individual towards their abilities, competencies, and position (Rosenberg et al., 1995). Regarding its relationship with the use of social networks, it has been shown that ascending social comparison negatively influences self-esteem (Schmuck et al., 2019).

Coping styles represent cognitive and behavioural efforts to reduce stress, involving an individual's psychological adaptation to those stressful stimuli (Lazarus & Folkman, 1984). People with attitudes and negative body image schemas may be more likely to engage in maladaptive coping strategies to help manage their body-related distress after engaging in biased social comparisons (Lewis-Smith et al., 2019). In addition, the additional stress caused using social networks, such as the leakage of personal information or comparisons with others (Vonk et al., 2019), causes a greater use of coping strategies focused on emotions (Wadsworth & Compas, 2002). The use of adaptive coping has been associated with increases in body satisfaction after exposure to thin ideal images (Wade et al., 2009).

The construct of well-being reflects the optimal experience and performance of human beings. In this research, two approaches coexist: (i) the hedonic perspective, called subjective well-being, which is defined in terms of achievement and pain avoidance, and focused on positive affect, such as happiness or on life satisfaction; (ii) the eudaimonic perspective, or the meaning and self-realization, defines well-being as the degree of fully functioning, and known as psychological well-being (Deci & Ryan, 2008). In general, exposure to content related to aesthetic ideals uploaded by others can have detrimental consequences for well-being (Kleemans et al., 2018). The presentation of self-ideals in social networks also seems to reduce the indicators of well-being (Jackson & Luchner, 2018); however, the honest self-presentation can increase it (Grieve & Watkinson, 2016).

This study aimed to jointly address insufficiently developed objectives so far in the research on the use that young people make of Instagram. In this way, the present research aims a) to explore whether people with divergent body sizes are likely to adopt different types of selfies, such as body-centered versus face-centered images; and b) whether body image and the appearance schemas mediates the relationship between the level of body exposure on Instagram and BMI. As such, we formulated the following hypotheses:

*H1*: Users with a lower BMI will be more willing to show their image on Instagram, while users with a higher BMI will be less willing to show their physical appearance.
*H2*: Participants with a lower BMI will present a higher positive body image and better psychological status -self-esteem, coping, and well-being- than those with a higher BMI.
*H3*: The level of whole-body self-exposure on Instagram is predicted by BMI and, in turn, it is predicted by a lower body image and by appearance schemas focused on defining oneself through appearance and engaging in grooming and appearance management behaviors.

## Method

### Participants

The present study had the participation of 687 Instagram users, 465 women (67.7%) and 222 men (32.3%) who were contacted through the university, between 2019 and 2021. A descriptive design, through surveys, chain-referral sampling, was used, with ex post facto cross-sectional samples. Participants ranged in age between 18 and 35 years old (*M* = 23; *SD* = 4.71), corresponding the distribution of Instagram users worldwide in July 2019 (Clement, 2019). According to the ranges of the BMI, 35 women and 7 men were underweighted, 303 women and 155 men were in a normal weight state, 94 women and 47 men were overweight, and 33 women and 13 men were obese. The average number of publications uploaded to Instagram was 130 images for women and 70 for men. Regarding the content of these publications, women tend to upload photos of content not related to people in 78.1%, only of their face in 63.4%, with their torso visible in 63.4% of the cases, and of their entire body in 61.9%. Whereas men tend to upload photos of content not related to people in 83.3%, only of their face in 81.1%, with their torso visible in 71.6%, and of their entire body in 59.5%.

### Materials

Survey about demographics and Instagram use—developed by the authors. It includes questions about demographics (age, gender, academic level, occupation) height and weight, were included. Additionally, several aspects about the Instagram use, was thoroughly assessed, based on the questionnaire for the use of Facebook (Meier & Gray, 2014). Concerning the exposure, that is, taking photos of themselves to be uploaded to Instagram, we have distinguished among four frequency levels (from almost never or never, to almost always or always) of body parts exposure on Instagram (Table 1). The internal consistency indexes of these items were accurate (α = .73).

**Table 1:** Exposure on Instagram

Items referring to user exposure on Instagram	Almost never or never	Rarely	Often	Almost always or always
I upload a photo of myself, only of my face	33.9%	35.2%	22.4%	8.4%
I upload a photo of myself, my waist and chest visible	38.1%	27.9%	27.4%	6.6%
I upload a photo of myself, all my body visible	33.8%	27.4%	28.2%	10.6%
I upload a photo of myself with friends and/or family	20.2%	29.7%	36.4%	13.7%
I upload a photo of a text or an image whose content is not related to people	58.7%	21.1%	13.5%	6.7%
I upload a story of myself	30.1%	27.7%	25.5%	16.7%
I upload a story of myself with friends and/or family	19.4%	26.2%	36.1%	18.3%
I upload a story of a text or an image whose content is not related to people	30.9%	25.3%	27.2%	16.6%

Body Appreciation Scale (BAS; Avalos et al., 2005). It consists of 13 items, whose factorial structure includes a single dimension of Positive Body Image, with adequate internal consistency and construct validity to study the positive aspects of body image. The questionnaire must be answered on a Likert scale, with five alternatives (1 = “I never do this”, 5 = “I always do this”). Scores on the BAS have been found to be internally consistent, with Cronbach’s alpha coefficient of .93.

Appearance Scheme Inventory-Revised (ASI-R; Cash & Grasso, 2005). It consists of 20 items that evaluate two facets of body image: (i) Self-Assessed Significance, made up of 12 items that examine the extent to which individuals define themselves through their physical appearance; (ii) Motivational Significance, made up of 8 items and assesses the degree to which people engage in behaviors for the care and management of their physical appearance. The questionnaire used a Likert scale with five alternatives (1 = “I completely disagree”, 5 = “I completely agree”). Scores on the ASI-R shown an accurate internal consistency in its two factors, Self-Assessed Significance (α = .88) and Motivational Significance (α = .85).

Rosenberg Self-Esteem Scale (RSES; Rosenberg, 1965). It consists of 10 items that express positive and negative thoughts about personal worth, to be answered on a 4-point Likert scale from 1 (strongly agree) to 4 (strongly disagree). Scores on the RSES have been found to be internally consistent, with Cronbach’s alpha coefficient of .64.

Brief Cope Inventory (COPE-28; Carver, 1997). It consists in the following subscales: Active Coping, Planning, Positive Reframing, Acceptance, Humor, Religion, Using Emotional Support, Self-Distraction, Denial, Venting, Substance, Behavioral Disengagement and Self-Blame. The items are presented in terms of the action and the response that people make on an ordinal scale with four alternatives (0 = “I never do this”, 3 = “I always do this”). The internal consistency indexes were accurate for most scales (Active Coping α = .64, Planning α = .65, Positive Reframing α = .63, Humor α = .78, Religion α = .78, Using Emotional Support α = .72, Denial α = .62, Substance α = .93, Behavioral Disengagement α = .66, and Self-Blame α = .65), except for Acceptance (.58), Self-Distraction (.53), and Venting (.39).

Positive and Negative Affect Scales (PANAS; Watson et al., 1988). It consists of 20 qualifiers: 10 measure positive emotions and 10 negative emotions that may be present in the individual during the last month. Every item is assessed using a Likert-type scale of seven scores ranging from 1 (very little) to 5 (a lot), depending on the presence of each emotion at that moment. The reliability through the Cronbach’s alpha coefficient-indicates a good internal consistency (Positive Affect, α = .88 and Negative Affect, α = .88).

Satisfaction with Life Scale (SWLS; Diener et al., 1985). It consists of 5 items that evaluate the individuals’ cognitive judgment about their global life satisfaction, comparing their life circumstances with a particular standard. Each item is answered on a scale that ranges from 1 (not at all satisfied) to 7 (very satisfied). The reliability through the Cronbach’s alpha coefficient indicates a good internal consistency (α = .88).

Subjective Happiness Scale (SHS; Lyubomirsky & Lepper, 1999). The SHS consists of 4 items with a 7-point response format: from 1 (not happy) to 7 (very happy). The reliability through the Cronbach’s alpha coefficient indicates a good internal consistency (α = .82).

Psychological Well-Being Scale (PWBS; Ryff, 1989). It measures the six dimensions proposed by the Ryff model: Self-acceptance, Positive Relationships with Others, Autonomy, Environmental Mastery, Purpose in Life and Personal Growth, through a 6-point Likert-type scale. Using a Likert scale with six alternatives (1 = “I completely disagree”, 6 = “I completely agree”). The scales showed good internal reliabilities, (Self-Acceptance α = .86, Autonomy α = .73, Environmental Mastery α = .66, Purpose in Life α = .82; and Personal Growth α = .64), except for Positive Relationships with Others (α = .04).

### Procedure

The evaluation battery has been administered electronically and includes the previously described questionnaires to analyze the variables in young Instagram users. The inclusion criteria in the study were: a) to be between 18 and 35 years old; and b) to have an active Instagram account. Participants were provided information on the conditions of the study and the ethical treatment of the data, proceeding to give their informed consent. At the beginning of the battery of questionnaires, the objectives of the study, its anonymous nature, and the use of the data only for research purposes were informed, thus requesting their personal consent to use this information for those purposes.

### Data Analysis

After computerizing the information in the SPSS 24.0, an ANOVA was performed to observe the possible differences between the four ranges of body mass index (underweight, normal weight, overweight, and obesity) for women and a Kruskal-Wallis test for men. Once it was known in which variables there were significant differences, *post hoc* contrasts were performed between these four BMI ranges, to find out between which subgroups these differences occurred.

Subsequently, correlational analyzes (Pearson's *r*) both for women and men, among the item *I upload a photo of myself, all my body visible*, as whole-body self-exposure, and the variables Body Image, Coping, Self-Esteem, Subjective Well-Being, and Psychological Well-Being, were performed, as well as, with the BMI and the following psychological variables: Body Image, Coping, Self-Esteem, Subjective and Psychological Well-Being.

In the correlation matrix, BMI only had a significant correlation with whole body self-exposure, but not with *I upload a photo of myself, only of my face*, as well as with Self-Assessed Significance. Because of this, we took these two variables out of the multiple mediation model.

Thus, we set out test multiple mediation models using BMI as the predictor, Whole-body self-exposure as the criteria and Positive Body Image and Appearance Schemas (Self-Assessed Significance and Motivational Significance) as the mediator based on previous findings.

The analysis was carried in R using lavaan. Considering that Whole-Body Self-Exposure was an ordinal variable, we used the polychoric correlation matrix and DWLS as the estimator to perform the mediation model.

## Results

To observe possible differences in the type of photographs uploaded to Instagram and in the psychological variables, depending on the different BMI ranges, we performed a one-way ANOVA for women and a Kruskal-Wallis test for men, due to the size of both subsamples. Having homogeneity of variances (Levene's test), the results between the four BMI ranges are shown in Table 2 for women and Table 3 for men.

**Table 2:** Women Comparison (One-Way ANOVA) on Exposure Variables and Psychological Adjust Among the Different Ranges of BMI

	*M* ± *SD*					
	Underweight	Normal weight	Overweight	Obese		
Variable	(*n* = 35)	(*n* = 303)	(*n* = 94)	(*n* = 33)	*F*_(3, 461)_	α
Self-Exposure						
I upload a photo of myself, my waist and torso visible	2.26 ± 1.01	2.14 ± 0.97	1.95 ± 1.04	1.73 ± 0.88	2.76	.042
I upload a photo of myself, my whole body visible	2.37 ± 1.03	2.24 ± 1.00	1.96 ± 0.96	1.76 ± 0.87	4.31	.005
I upload a photo of a text or an image whose content is not related to people	1.86 ± 0.97	1.64 ± 0.93	1.78 ± 1.01	2.18 ± 1.58	3.48	.016
I upload a story of myself with friends or family	3.08 ± 0.98	2.71 ± 0.94	2.56 ± 0.98	2.46 ± 1.18	3.20	.023
Body Image:						
Positive Body Image	49.29 ± 9.85	47.01 ± 10.20	43.23 ± 10.76	34.12 ± 12.14	18.19	.000
Self-Assessed Significance for Appearance Schemas	27.69 ± 9.12	26.81 ± 7.72	29.19 ± 8.23	30.21 ± 9.54	3.33	.019
Motivational Significance for Appearance Schemas	24.37 ± 6.41	23.35 ± 5.37	23.67 ± 5.51	20.58 ± 5.85	3.27	.021
Coping						
Active Coping	6.80 ± 1.05	6.52 ± 1.16	6.65 ± 1.21	5.82 ± 1.61	4.75	.003
Planning	6.66 ± 1.16	6.08 ± 1.33	6.39 ± 1.32	5.67 ± 1.57	4.41	.004
Positive Reframing	6.34 ± 1.37	5.90 ± 1.45	6.15 ± 1.38	5.21 ± 1.49	4.53	.004
Self-Blame	5.40 ± 1.63	5.39 ± 1.54	5.86 ± 1.62	6.09 ± 1.72	3.72	.012
Well-being						
Life Satisfaction	23.86 ± 6.75	24.15 ± 5.97	23.48 ± 7.17	19.09 ± 8.83	6.00	.001
Purpose in Life	22.83 ± 4.74	22.08 ± 4.55	22.96 ± 4.99	20.30 ± 5.85	2.81	.039

**Table 3:** Men Comparison (Kruskal Wallis) on Exposure Variables and Psychological Adjust Among the Different Ranges of BMI

	*M* ± *SD*					
	Underweight	Normal weight	Overweight	Obese		
Variable	(*n* = 7)	(*n* = 155)	(*n* = 47)	(*n* = 13)	χ^2^(3)	α
Self-Exposure						
I upload a photo of myself, my waist and torso visible	2.00 ± 0.82	2.03 ± 0.88	1.57 ± 0.85	1.46 ± 0.52	14.27	.003
I upload a story of myself	2.14 ± 0.69	2.21 ± 1.01	1.96 ± 1.04	1.54 ± 0.88	7.79	.050
I upload a story of a text or an image whose content is not related to people	2.43 ± 0.98	2.15 ± 1.01	1.53 ± 0.88	2.08 ± 1.18	16.98	.001
Body Image						
Positive Body Image	42.14 ± 13.61	51.62 ± 8.96	46.70 ± 9.90	40.38 ± 7.56	26.11	.000
Motivational Significance for Appearance Schemas	21.29 ± 3.09	22.48 ± 6.00	21.43 ± 5.73	17.46 ± 5.16	9.67	.022
Coping						
Active Coping	6.14 ± 0.69	6.37 ± 1.28	5.96 ± 0.98	5.69 ± 1.32	8.97	.030

To analyze among which groups of BMI ranges (underweight, normal weight, overweight and obesity) these differences were found, *post hoc* contrasts were made, using the Games-Howell Test, because our design is not orthogonal. We have found significant differences in:

- *Self-Exposure*: “I upload a photo of myself, my whole body visible” between underweight and obesity (*p* = .047, *d* = .640), normal weight and obesity (*p* = .024, *d* = .512); and “I upload a story of myself with friends or family” between underweight and overweight (*p* = .045, *d* = .572).
- *Body Image*: Positive Body Image between underweight and overweight (*p* = .018, *d* = .587), underweight and obesity (*p* < .001, *d* = 1.372), normal weight and overweight (*p* = .016, *d* = .361), normal weight and obesity (*p <* .001, *d* = 1.150), overweight and obesity (*p* = .002, *d* = .794). Motivational Significance between overweight and obesity (*p* = .050, *d* = .544).
- *Coping*: Active Coping between underweight and obesity (*p* = .023, *d* = .721), overweight and obesity (*p* = .045, *d* = .583). Planning between underweight and normal weight (*p* = .043, *d* = .465), underweight and obesity (*p* = .024, *d* = .717). Positive Reframing between underweight and obesity (*p* = .010, *d* = .790), overweight and obesity (*p* = .014, *d* = .655).
- *Well-Being*: Life Satisfaction between normal weight and obesity (*p* = .014, *d* = .671).
- To observe among which groups of BMI ranges (underweight, normal weight, overweight and obesity) these differences were found, *post hoc* contrasts were made, using the test of Mann-Whitney. We have found significant differences in:
- *Self-Exposure*: “I upload a photo of myself, my waist and torso visible” between normal weight and overweight (*z* = -3.26, *p* = .001, *d* = .532), normal weight and obesity (*z* = -2.26, *p* = .024, *d* = .789); “I upload a story of myself” between normal weight and obesity (*z* = -2.45, *p* = .014, *d* = .707); and “I upload a story of a text or an image whose content is not related to people” between underweight and overweight (*z* = 2.62, *p* = .009, *d* = .966); normal weight and overweight (*z* = -0.98, *p* < .001, *d* = .655).
- *Body Image*: Positive Body Image between normal weight and overweight (*z* = -3.21, *p* = .001, *d* = .521), normal weight and obesity (*z* = -4.06, *p* = .000, *d* = 1.356), overweight and obesity (*z* = -2.31, *p* = .021, *d* = .718). Motivational Significance between normal weight and obesity (*z* = -2.92, *p* = .003, *d* = .897), and overweight and obesity (*z* = -2.19, *p* = .028, *d* = .728).
- *Coping*: Active Coping between normal weight and overweight (*z* = -2.55, *p* = .011, *d* = .360).

Subsequently, correlational analyzes (Table 4) have been carried out, both for women and men, between the item *I upload a photo of myself, all my body visible*, as Whole-Body Self-Exposure, and the ranges of BMI, Body Image, Coping, and Personal Well-Being (except for Positive Relations due to its low internal consistency).

**Table 4:** Correlational Analyses of Self-Exposure of Whole Body on Instagram, BMI Ranges, Body Image and Psychological Variables

	Whole-Body Self-Exposure (*r*)	
Variable	Women	Men
BMI ranges	-.162***	-.144
Body Image		
Positive Body Image	.257***	.148*
Motivational Significance for Appearance Schemas	.211***	.253***
Coping		
Emotional Support	.114*	.099
Active Coping	.087	.138*
Denial	-.040	.141*
Self-esteem	.203***	.070
Subjective Well-Being		
Happiness	.228***	.115
Positive Affect	.061	.188**
Satisfaction with Life	.146**	.153*
Psychological Well-Being		
Self-Acceptance	.192***	.185**
Purpose in Life	.167***	.165*
Environmental Mastery	.157**	.141*

*p ≤ .05. **p ≤ .01. ***p ≤ .001.

**Figure 1:**
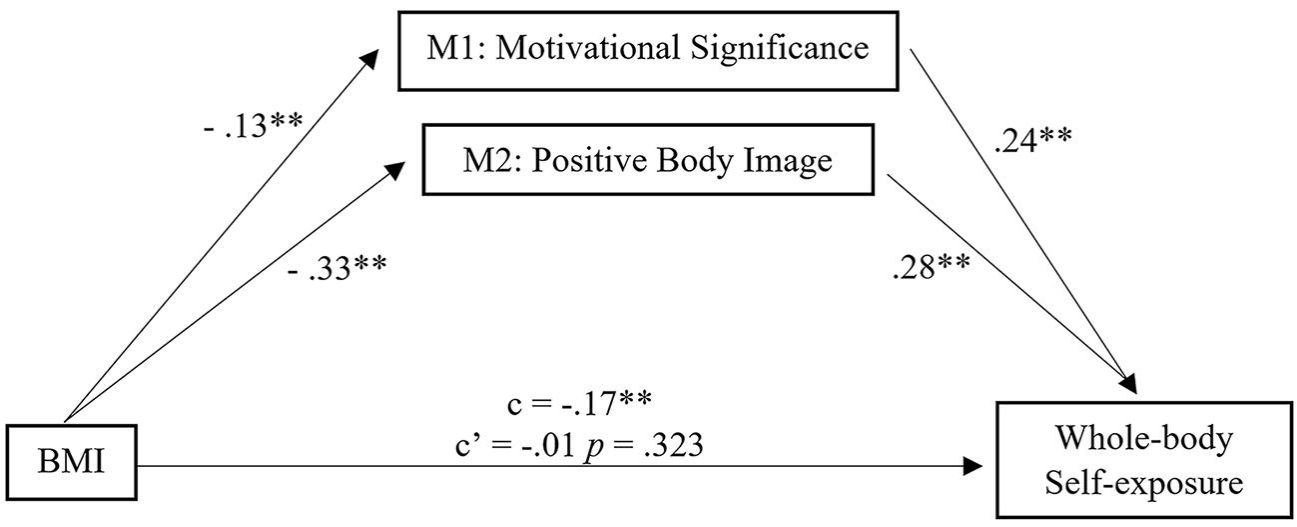
Mediation Model for Women *p ≤ .05. **p ≤ .01. ***p ≤ .001.

Due to the significant correlation between Whole-Body Self-Exposure and BMI only among women, we performed a test multiple mediation model using BMI as the predictor, Whole-Body Self-Exposure as the criteria, and Positive Body Image and Motivational Significance as the mediators just for the women sample, presented in the Figure 1.

BMI was a significant predictor of Whole-Body Self-Exposure (*b* = -.040, *SE* = .01, *p* < .001). The standardized coefficient of -.17 reflects the direct effect of BMI on Whole-Body Self-Exposure, the c path in the model. Both paths from the BMI and the mediators were significant (*p* < .001). The paths from the mediators to Whole-Body Self-Exposure were also significant (*p* < .001).

After controlling the effects of the mediators, BMI was no longer a significant predictor of Whole-Body Self-Exposure (*b* = -0.10, *SE* = .01, *p* = .323). The standardized regression coefficient for this path is -0.012. The total indirect effect was significant (-0.040, *SE* = 0.012, *p* < .001).

## Discussion

The present research has been focused on the link between Body Mass Index and Self-Exposure on the Internet, that particularly young people make on Instagram, and their relation to certain psychological variables. In our sample, we can see that women post photos on Instagram almost twice as often as men. Women post more selfies of their faces and torso than men, while men post more selfies of their full body than women, and women post more content unrelated to themselves, which is in accordance with the results found by Haferkamp et al. (2012) and Simerson (2016).

Regarding the contrasts between the ranges of BMI (underweight, normal weight, overweight and obesity), the results have shown significant differences in the exposure of the body itself on Instagram, in both genders. There were differences among women in those photos that involve showing more body, not relating to other people, or those in which they share stories with other people on them. In this sense, slim women post more body-centered photos, and post less non-person-related content. Regarding men, there are also significant differences in sharing the torso and waist in their photos and sharing stories of themselves or content no related with people, being the lower range of BMI who carries out these behaviors. These findings confirm our first hypothesis and are in line with Haferkamp et al. (2012) who found that women are more likely to post more parts of their body on social networks. People with higher BMIs may feel more uncomfortable showing their whole body on the Internet (Couture Bue, 2020).

There are also significant differences on body satisfaction in both genders. Women showed less Positive Body Image and Motivational Significance as their BMI increase and the opposite way in Self-Assessed Significance. This could suggest that thin women and regular-weight men tend to adopt behaviors for the care and management of their physical appearance. In terms of Self-Assessed Significance, heavier women were shown to define themselves through their physical appearance. This may be explained by the fact that heavier women might engage in weight-reducing behaviors, such as dieting or exercise. Men tended to have a better Positive Body Image on the middle ranges of BMI and a worse Motivational Significance on the highest range of BMI. So, men tend to be more concern about their appearance, whether they are very thin or obese. But, when they are obese, they don’t try to fix their appearance likewise women do. These results may be explained by the fact that men with a lower BMI wanted to be more muscular, with BMI being a predictor of the drive for muscularity (Daniel & Bridges, 2010). All these findings are also in line with the research from Butkowski et al. (2019), which postulated that people with a higher BMI usually report greater body dissatisfaction and a drive for thinness; and the results from Ridgway and Clayton (2016) who found that people who posted more photos on Instagram showed more frequently higher body satisfaction.

Concerning the rest of psychological variables, there are also significant differences between genders and their bodily exposure in coping. Women tend to use more adaptive coping strategies—such as Active Coping or Planning—the thinner they are. On the other hand, women with higher BMI use more maladaptive coping such as Self-Blame in the higher ranges of BMI. For men, there is also a use of adaptive coping in the lower ranges of BMI, but no differences in maladaptive coping. These findings confirm the first hypothesis, and they are in line with other research (Pinkasavage et al., 2015; Sarwer et al., 2005), which showed a greater use of maladaptive ways of coping and lower positive body image in people with higher BMI. Lastly, the significant differences in Well-Being are only seen among women, so that, the lesser weigh, the more Life Satisfaction, and Purpose in Life. Results that are according to preliminary research relating high levels of BMI with worse well-being and quality of life (Sarwer et al., 2005; Wilson et al., 2013).

Through the test of our model, we found the following results among women: (i) a negative relationship between BMI and Positive Body Image and Motivational Significance; (ii) a negative relationship between BMI and Whole-Body Self-Exposure; and (iii) a positive relationship between the mediating variables and Whole-Body Self-Exposure. Additionally, our model presents a full mediation in which there is not a direct relation between BMI and the exposure of the entire body on Instagram. The relationship between BMI and Whole-Body Self-Exposure exists solely through appearance care and appearance management behaviors, and Positive Body Image. Some studies indicate that individuals with high BMI avoid exposing themselves to situations where their appearance may be criticized for fear of being negatively judged by others (Levinson & Rodebaugh, 2012), and that individuals with high BMI tend to report negative body image and body shape concerns (Ahadzadeh et al., 2018; Holsen et al., 2012). However, our study shows a superior value of positive body image and motivational importance in the decision to expose one’s body in networks. Thus, weight would be a variable that is not directly associated with self-exposure; so, people with a better valuation of their own appearance would be those who would be willing to show more of their body on the Internet.

The mediating effect of Positive Body Image and Motivational Significance supports the claim that these variables are an important resource in explaining the negative relationship between BMI and self-body exposure on Instagram. These results are consistent with studies demonstrating the importance of positive body image for self-body acceptance. A tentative explanation of these findings could be that women with a positive body image are more likely to report fewer negative feelings towards their bodies and appearance; or even the reverse link, given the cross-sectional character of this study. These individuals may show less fear of others’ judgement of their body regardless of their weight, and thus show more parts of their body on social networks (Cash, 2008; Sánchez-Carracedo et al., 2004).

### Limitations and Future Directions

These findings should be taken with caution, given some limitations of the research itself, namely: the survey about Instagram use, elaborated by the authors, which has been carried out on this research, so it has not undergone a validation procedure. In addition, the use of abbreviated questionnaires may affect internal consistency and the statistical results obtained. On the other hand, the sample is made up of 68.2% female and 31.8% male, so future research should promote a greater sample collection of males. Finally, the subgroups of BMI are not balanced, showing substantial differences between the amount of people with normal weight (63.3%), overweight (31.8%), people with underweight (6.0%), and obesity (6.8%); since the participation of people in the most extreme ranges of the BMI should be promoted.

In our opinion, the present study captures a reality insufficiently considered in previous research, focusing on the implications that BMI, Positive Body Image and Appearance Schemas may have on the personal conceptions of young people who use the Internet. The role that Positive Body Image and Motivational Significance play in explaining the negative relationship between BMI and exposure of one’s body on Instagram demonstrates the importance of cultivating this appreciation of one’s body in order to improve the well-being of weightier individuals and enable them to be more inclusive in society. We understand, in this way, that a path has been opened for future research on this topic to understand and, as far as possible, alleviate and avoid the psychological consequences that this situation can cause.

### Conclusions

This study has analyzed the different degrees of exposure of the body in the photos that young people upload to Instagram according to gender and based on different BMI ranges. The results obtained have shown that people with a higher body weight tend to upload fewer photos in which their body is visible -medium and full body shots. In addition, they show greater body dissatisfaction, maladaptive coping strategies and, especially women, poorer well-being. On the other hand, the importance of positive body image and appearance management behaviors in posting full-body photos on Instagram seems to be crucial.

Therefore, it is not only necessary to pay more attention to the pressure that these people receive from social networks that prevent them from showing their bodies for fear of exposure and physical competition with their peers, but also to the appreciation of their own bodies that these people have. Thus, this work highlights the importance of promoting a positive body image for body acceptance and body confidence, so that people with a high BMI feel confident enough to show their body to others. Furthermore, the findings can contribute to a more diverse and inclusive society, which can help others to accept their own bodies as well.
